# Effects of statins beyond lipid-lowering agents in ART-treated HIV infection

**DOI:** 10.3389/fimmu.2024.1339338

**Published:** 2024-04-09

**Authors:** Vikram Mehraj, Jun Chen, Jean-Pierre Routy

**Affiliations:** ^1^ Research Centre McGill University Health Centre, Montreal, QC, Canada; ^2^ Department of Infectious Diseases and Immunology, Shanghai Public Health Clinical Center, Shanghai, China; ^3^ Chronic Viral Illness Service and Division of Hematology, McGill University Health Centre, Montreal, QC, Canada

**Keywords:** statins, HIV, cardiovascular diseases, inflammation, immune activation, REPRIEVE randomized clinical trial

## Abstract

Antiretroviral therapies (ART) have reduced human immunodeficiency virus (HIV) infection-associated morbidity and mortality improving the life of people with HIV (PWH). However, ART lead to residual HIV production, which in conjunction with microbial translocation and immune dysfunction contributes to chronic inflammation and immune activation. PWH on ART remain at an increased risk for cardiovascular diseases (CVDs) including myocardial infarction and stroke; which in part is explained by chronic inflammation and immune activation. Lifestyle factors and certain ART are associated with dyslipidemia characterized by an increase of low-density lipoprotein (LDL), which further contributes in the increased risk for CVDs. Lipid-lowering agents like statins are emerging as immune modulators in decreasing inflammation in a variety of conditions including HIV. The international randomized clinical trial REPRIEVE has shed light on the reduction of CVDs with statin therapy among PWH. Such reports indicate a more than expected benefit of statins beyond their lipid-lowering effects. Bempedoic acid, a first-in-class non-statin LDL-lowering drug with immune modulatory effects, may further aid PWH in combination with statins. Herein, we critically reviewed studies aimed at lipid-lowering and immune-modulating roles of statins that may benefit aging PWH.

## Introduction

Antiretroviral therapies (ART) control viral replication without curing HIV infection and need to be taken for long-term. ART significantly improve the quality of life of people with HIV (PWH) by improving immune function, reducing morbidity and increasing survival (1). However, the risk for inflammatory non-AIDS events such as cancer, neurocognitive disorders, liver dysfunction and cardiovascular diseases (CVDs) remains elevated among PWH on ART (1–4). Shah et al. did a meta-analysis recently and confirmed previous findings that PWH are at a greater risk for the development of CVDs in comparison to people without HIV (5). In part, such elevated risk in PWH is explained by chronic inflammation and immune activation and a higher proportion having traditional risk factors such as smoking, diabetes, and dyslipidemia (6, 7). Factors associated with inflammation and immune activation among PWH include HIV RNA and proteins produced on ART (8), gut damage and subsequent microbial translocation coupled with gut microbial dysbiosis and co-infections such as hepatitis C virus (HCV) and cytomegalovirus (CMV) (9, 10). Increased levels of several markers of inflammation and immune activation such as C-reactive protein (CRP), interleukin (IL)-6 and soluble CD14 (sCD14) have been linked with CVD events among PWH (10). A widely studied systemic indirect marker of microbial translocation is sCD14, a monocyte activation marker which has also been associated with mortality among PWH (9, 11). Zidar et al. reported among 54 PWH versus 24 controls an association of sCD14 with oxidized low density lipoprotein (ox-LDL) (12), which is also considered a predictor of CVDs (13). We reported a correlation of sCD14 with soluble suppressor of tumorigenicity 2 (sST2) (14), a marker of cardiopulmonary dysfunction and fibrosis (15, 16), in the context of immune activation among PWH. In addition, 1,3-β-D-Glucan (βDG), a marker of microbial translocation of fungal origin contributes in chronic immune activation and has also been associated with CVDs (17, 18).

Dyslipidemia leads to CVDs and has been linked with inflammation and boosted protease inhibitors-based ART among PWH (19, 20). However, such risk is reduced with the use of integrase strand transfer inhibitor (INSTI)-based ART regimens that are increasingly being prescribed in two-drug (2-DR) or three-drug (3-DR) regimens (21–23). Increased risk for CVDs is evident among PWH versus general population even after taking into account the traditional risk factors such as smoking, physical inactivity and diabetes (4, 24, 25). Such risk is reduced with the use of statins that not only reduce dyslipidemia but also act as an immune modulator (7). Despite these benefits, statin utilization among PWH remains relatively low in part due to potential adverse effects and interactions with ART (26, 27). In addition, there is a dearth of large-scale intervention studies focusing on optimal statin utilization among PWH. The results from the NIH co-funded, multi-center Randomized Trial to Prevent Vascular Events in HIV (REPRIEVE, NCT02344290) showed that a fixed daily dose of 4mg of pitavastatin calcium vs. placebo (in a 1:1 randomization) reduced by 35% the incidence of major CVD events over a median follow-up of 5.1 years (28). Such REPRIEVE results showing significant reduction of CVD events with statin use among PWH with low risk profile are consistent with the importance of an inflammatory aetiology for much of the CVD risk in this population. Among adverse events, muscle-related symptoms and incident diabetes mellitus (DM) respectively were 1.7 and 1.4 times more likely to be reported in pitavastatin group compared to placebo. Overall, a large sample size and a multi-ethnic study population recruited from 12 countries across continents make REPRIEVE a unique seminal study. This review aims to summarize findings from studies including REPRIEVE that evaluated statins as lipid-lowering agents and as immune modulators among people with or without HIV.

## Overview of statins and their mechanism of action

Mevastatin or compactin was the first member of statins administered, which was isolated in Japan in 1976 by a biochemist, Akira Endo, from a fungal culture medium of *Penicillium citrinum* (29). However, the development of mevastatin was discontinued, as the drug caused lymphoma in dogs that received extremely high doses (which was about 200 times the dosage that would be used in humans). Mevastatin discovery was followed by lovastatin and simvastatin in the 1980s, both of these are also fungal metabolites. Later, the chemically modified next generation of statins such as pravastatin and fluvastatin were approved, which were comparatively more bioavailable. Rosuvastatin represents the new generation of hydrophilic statins with a longer half-life and a better potency (30). Currently, there are seven FDA approved medications in the class of statins that are commonly used, namely: atorvastatin, fluvastatin, pitavastatin, lovastatin, simvastatin, rosuvastatin, and pravastatin. These can also be grouped as natural statins such as lovastatin, simvastatin and pravastatin; and synthetic ones such as rosuvastatin, fluvastatin, atorvastatin and pitavastatin (31). Conflicting results have been reported on the superiority of hydrophilic or lipophilic statins in preventing CVDs (32). Statins are orally administered, well-tolerated and are generally considered a safe medication. Liver is the primary site of statin metabolism, while excretion mainly occurs via urinary tract.

**Figure 1:**
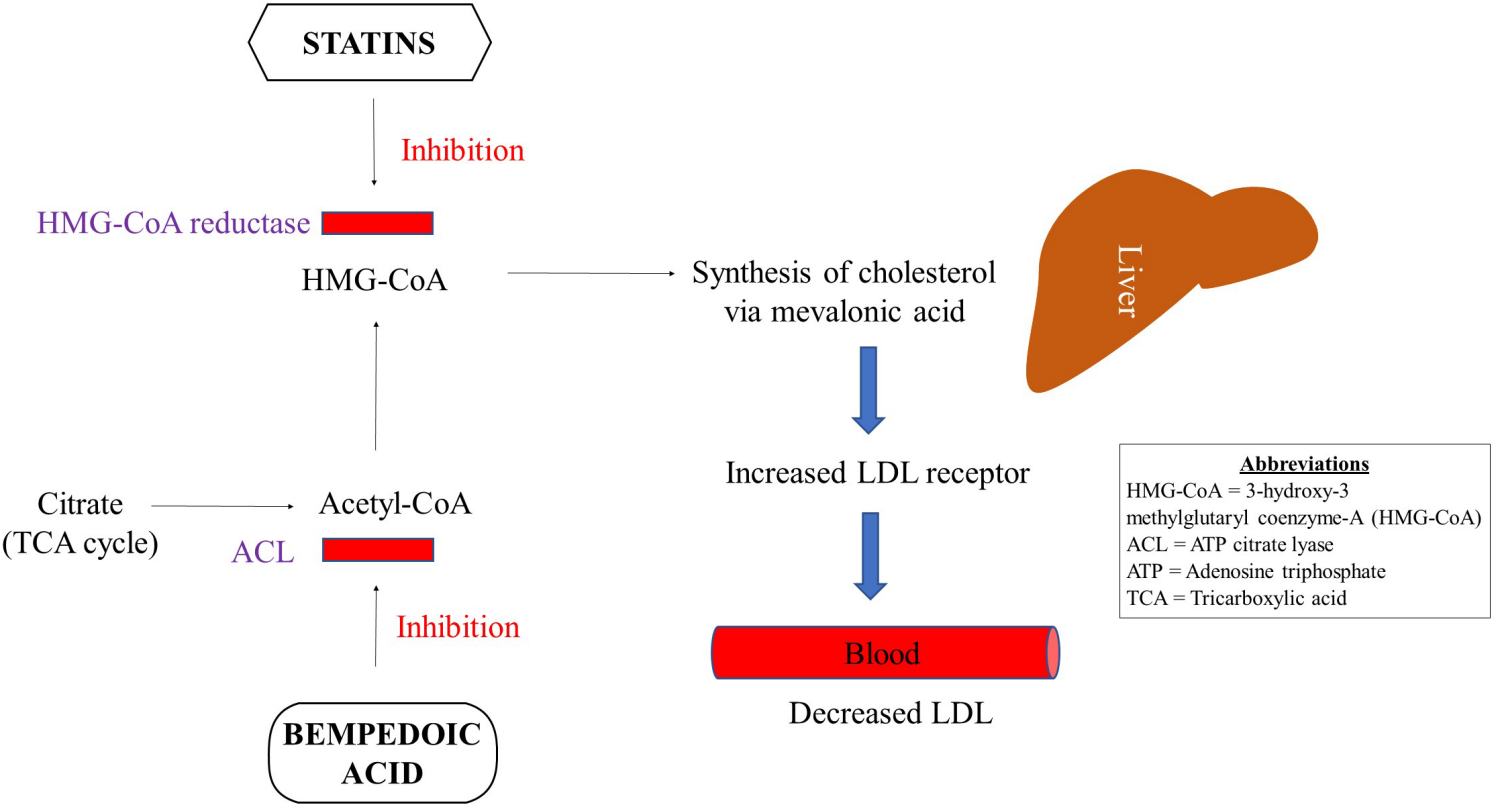
Schematic overview of Statin and Bempedoic acid converging mechanism of action.

**Figure 2:**
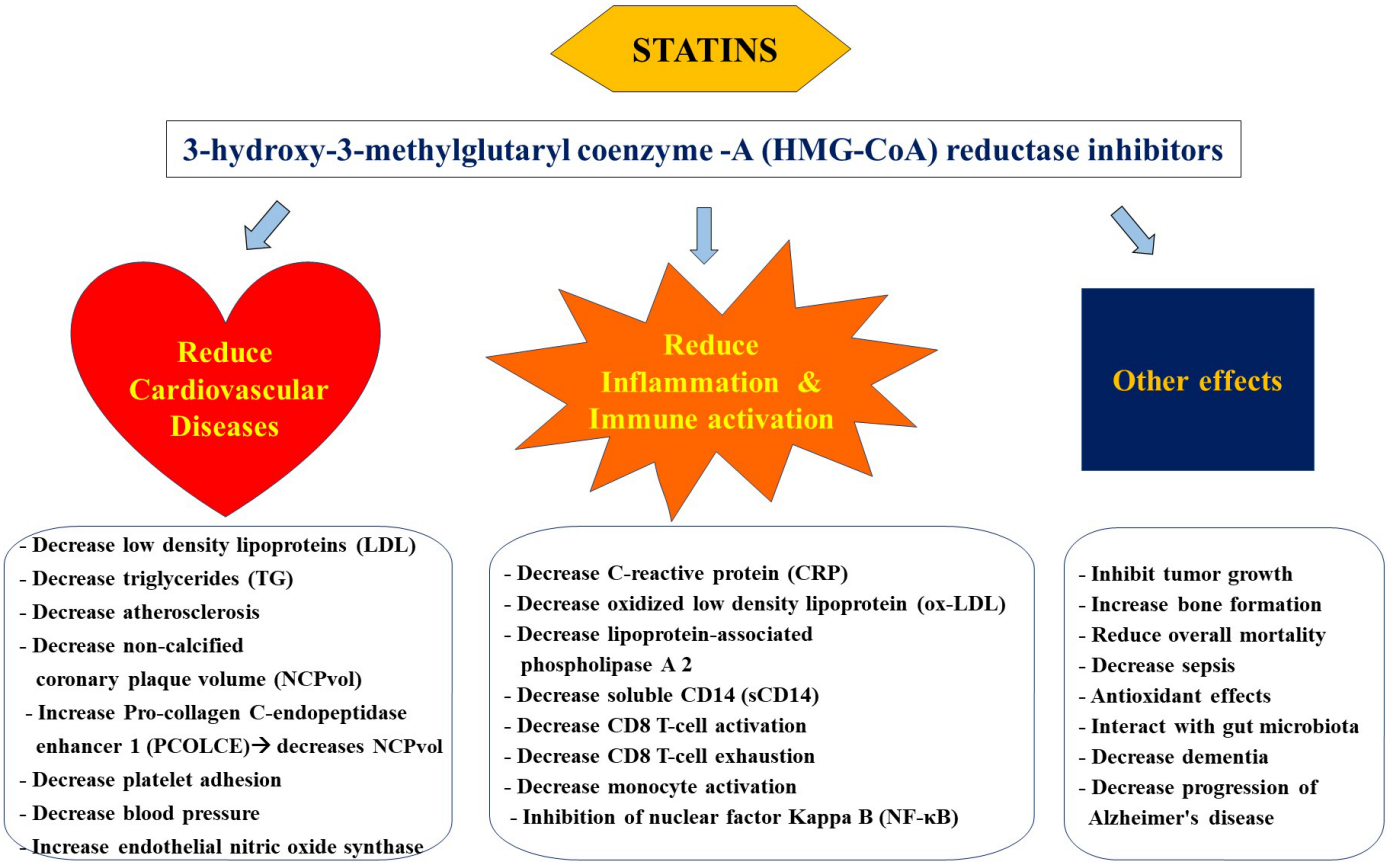
Pleiotropic effects of statins.

Statins regulate the concentration of plasma lipoproteins by inhibiting 3-hydroxy-3-methylglutaryl coenzyme-A (HMG-CoA) reductase at the proximal end of the mevalonate pathway (33) **Figure 1**. Inhibition of this rate-limiting step in the mevalonate pathway results in decreased endogenous production of cholesterol via overexpression of LDL receptors in the liver followed by a reduction in LDL particles in the periphery. Statins are increasingly considered a group of pleiotropic medications by being HMG-CoA reductase inhibitors that modulate the mevalonate pathway which is involved in a variety of functions such as cell proliferation and signaling, and platelet activation (34–36). Parihar and colleagues have recently reviewed the mechanism of action of statins in the context of anti-microbial therapy (30). Besides decreasing the risk for CVDs, statins are featured as immune modulatory medications that mainly decrease inflammation and immune activation (33, 37, 38) **Figure 2**.

Inflammation is mediated by the transcription regulator nuclear factor Kappa B (NF-κB) (39). Statins are involved in the inhibition of NF-κB activity by a variety of mechanisms ranging from: inhibition of Rho which is required for NF-κB activation and induction of kruppel like factor 2 (KLF2) which also suppresses NF-κB activation. Panigrahi and colleagues further demonstrated *in vitro* the protective effects of simvastatin on human aortic endothelial cells against KLF2 down-regulation induced by bacterial products and ox-LDL (40). Such functions highlight anti-inflammatory role of statins reviewed later in this manuscript.

## Statins and CVDs

Statins constitute first-line therapy for the reduction of LDL and triglycerides in circulation and are known to decrease the risk for CVDs in the general population (41). The cholesterol treatment trialists collaboration further estimated a reduction in CVDs of 22% in proportion to each decrease in LDL of 38.7 mg/dL (42). Among persons with increased levels of C-reactive protein (CRP), a marker of inflammation, the Justification for the Use of Statin in Prevention: An Intervention Trial Evaluating Rosuvastatin (JUPITER) led by Ridker et al. reported a more than expected reduction of CVDs with the use of rosuvastatin (43). This randomized clinical trial (RCT) included 17,802 apparently healthy persons with negative history of CVDs coupled with LDL levels below 130 mg/dL, and reported that 20 mg daily rosuvastatin reduced systemic CRP levels by 37% and LDL cholesterol by 50%, leading to a reduction in the incidence of CVDs such as myocardial infarction, stroke and unstable angina (43). In addition, all-cause mortality was also significantly reduced with the use of rosuvastatin. Among adverse events, the trial reported that the statin group had a higher incidence of diabetes but did not have a significant increase in muscle weakness or cancer. Along these lines, several studies have reported statins to reduce systemic levels of CRP, which is widely used as a predictor of CVDs (44, 45). Such results suggest implication of statins beyond lipid-lowering agents in the context of inflammation.

Wong and colleagues analyzed the National Health and Nutrition Examination Survey and estimated that despite statin use up to 80% of patients did not achieve an optimal LDL level (46). They attributed such sub-optimal statin response to less potent statin use, higher LDL levels at the initiation of therapy, aggressive LDL treatment goals or poor compliance. Therefore, alternate therapies or statin in combination with another medication such as bempedoic acid has been suggested for patients who respond poorly to statin monotherapy (47). Treatment non-compliance due to statin associated muscle-related adverse effects also prompts the use of alternate treatment options such as ezetimibe and bempedoic acid, both of which can also be used in combination with statins. Of note, bempedoic acid is a medication with minimal side effects owing to its localized gut effects. Like statins, bempedoic acid is increasingly being reported to have anti-inflammatory properties coupled with improved glucose metabolism (47). Bempedoic acid has been shown to have a converging mechanism of action with that of statins (**Figure 1**), and is briefly discussed below.

## Bempedoic acid: an emerging statin add-on

In 2020 FDA approved bempedoic acid (8-hydroxy-2,2,14,14-tetramethylpentadecanedioic acid), a novel first-in-class non-statin oral medication for the treatment of adults with atherosclerotic cardiovascular disease (ASCVD) or heterozygous familial hypercholesterolemia (HeFH) who did not achieve optimal LDL reduction on statins (48, 49). Bempedoic acid is known to reduce the levels of LDL in blood by inhibiting adenosine triphosphate (ATP) citrate lyase (ACL) in the metabolic synthesis pathway upstream of statins (47) (**Figure 1**). ACL is an important enzyme that connects carbohydrate to lipid metabolism. Bempedoic acid is a prodrug that is converted to its active form by acyl-CoA synthetase 1, which mainly is a hepatic enzyme, and is not expressed in the skeletal muscle. Therefore, bempedoic aced is less likely to cause muscle-related adverse effects (50). Additional benefits of bempedoic acid include activation of AMP-activated protein kinase (AMPK) that confers improved insulin sensitivity. Bempedoic acid is also recently shown to have anti-inflammatory properties by decreasing CRP level via activation of AMPK (47).

Bempedoic acid is prescribed in a single daily oral dose of 180mg which is generally well-tolerated. When given in combination with statins and/or ezetimibe in comparison to placebo, bempedoic acid significantly reduced LDL and CRP in patients with or at risk for ASCVD or HeFH (51). Ray et al. showed in a RCT involving 2230 patients with ASCVD and/or HeFH, with LDL ≥70mg/dl and receiving maximally tolerated statin dose that further reduction in LDL was achieved with the use of bempedoic acid versus placebo (49). They showed a reduction in LDL by 19.2mg/dl was achieved following 12 weeks of daily 180 mg dose of bempedoic acid. Both adverse events and serious adverse events were comparable in both arms of the trial with the exception of almost four times higher incidence of gout in the treatment arm. Bempedoic acid is also known to enhance the plasma levels of statins such as pravastatin and simvastatin and may require patient monitoring (47). In addition, it also offers a safer alternate in patients who are unable to tolerate statins. Therefore, it represents a promising medication in the context of HIV infection as a statin add-on or as an independent lipid-lowering and immune modulatory agent. Among ART-treated PWH on statins with poor treatment compliance owing to undesirable statin effects such as muscle issues, bempedoic acid could be a better option once evaluated for its safety and efficacy in large-scale randomized clinical trials.

## Statin use among PWH

Improvements in ART coupled with its early initiation are leading the evolution of HIV infection into a chronic condition with increased risks for co-morbidities in comparison to the general population. CVDs including myocardial infarction, unstable angina and stroke constitute the major portion of such co-morbidities and are overrepresented among PWH even after years being treated with ART. Shah et al. estimated in 2018 the global prevalence of HIV-associated CVDs to have tripled over the past two decades in a meta-analysis of 80 studies representing about 0.8 million participants and 3.5 million person-years of follow-up (5). HIV itself and associated chronic immune activation and inflammation jointly contribute to the risk of CVDs besides traditional factors such as smoking, diabetes and dyslipidemia (52). Ladapo et al. reported a lower prescription of statins among PWH versus their HIV-uninfected counterparts in a survey of nationally representative sample of adults at high risk for CVDs in the United States (26). Similarly, Wu et al. reported among PWH who met statin prescription criteria, only about 39% were actually prescribed statins (53). Statins among PWH are primarily prescribed to decrease dyslipidemia; thereby, reducing the risk for CVDs. However, Phan et al. reported a lack of association between statin use and carotid atherosclerosis progression or total mortality among PWH at high risk for CVDs (54). The authors further discussed the limitations of their study such as an observational study design, a small sample size, an overall small number of deaths and an overall small number of statin users which may have influenced their findings.

Grinspoon et al. recently reported results from much awaited REPRIEVE study, a phase 3 randomized controlled cosmopolitan clinical trial, largest ever conducted in PWH from March 2015 to July 2019 at 145 sites in 12 countries (28, 55, 56). Among 7,769 study participants 40-75 years of age, ART-treated, without CVD, renal or liver diseases, and with CD4 T-cell count > 100 cells/ul, nearly half were randomly assigned 4mg per day of pitavastatin calcium orally, while the rest were given an identical placebo. The median age of the study participants was 50 years and they did not have any past medical history of ASCVDs and were at a low to moderate risk of such conditions (57). The ASCVDs risk score was assessed by the 2013 American College of Cardiology/American Heart Association criteria (58). The trial was stopped earlier than planned as the statin therapy showed clear benefits which outweighed risks after a median follow-up of 5.1 years. The statin group in comparison to placebo had significantly lower incidence of major CVD events (4.8 vs 7.3 per 1000 person-years) with a hazard ratio of 0.65 (95% CI: 0.48-0.90). However, the intervention group also developed muscle-related symptoms and incident DM 1.7 and 1.4 times respectively, more than the placebo group, which remain lower than expected. Of note, pitavastatin comparatively remains less accessible owing to being expensive under patent. Along these lines, the extension of study findings to other statins remains a study limitation until determined, as also noted by Vergallo and Patrono (59). Grinspoon et al. further discuss that pitavastatin will soon be off-patent paving the way for the availability of its cheaper generic forms (60). In a mechanistic sub-study of REPRIEVE to evaluate the biological pathways implicated in statin response, using a targeted discovery proteomics approach among 542 participants, pitavastatin versus placebo was associated with a reduction in non-calcified plaque via Pro-collagen C-endopeptidase enhancer 1 (PCOLCE or PCPE-1), the rate-limiting enzyme in collagen deposition in interstitial tissue (61). Of note, changes in non-calcified coronary plaque volume were independent of changes in LDL. However, in another analysis Grinspoon et al. did not find an association between non-calcified or total coronary plaque volume and inflammatory markers in a representative sample of 604 study participants (62). Taken together, REPRIEVE is a benchmark study among PWH owing to its global representation, large multi-ethnic sample, strong study design and a prominent team of investigators.

Lo and colleagues reported a reduction of non-calcified coronary plaque volume, LDL and lipoprotein associated phospholipase 2 (LAP-A2) with one year use of atorvastatin in comparison to placebo in a small RCT with 40 ART-treated PWH with subclinical atherosclerosis (63) (**Table 1**). Several RCTs have further assessed the role of statins in decreasing inflammation, and LDL among treated PWH, as summarized in **Table 1** (28, 63–72). However, in comparison to the general population, a smaller decrease in LDL and triglycerides is achieved by the use of statins in PWH (73). Such sub-optimal statin response among PWH could partly be explained by co-administration with different ART regimens, different statin dosages, compliance, or the complexity of dyslipidemia in this population.

**Table 1:** Studies evaluating therapeutic role of statins among people with HIV (selected).

Study Reference	Study population	Type of statin used	Major Findings	Comments
Grinspoon et al. *N Engl J Med.* 2023	n=7769 PWH on ART with a low-to-moderate risk of cardiovascular disease Median age = 50 years	Pitavastatin vs placebo	Pitavastatin 4mg daily over 5.1 years median follow-up decreased the risk for major cardiovascular event (hazard ratio = 0.65; 95% CI=0.48-0.90; p=0.002)	Experimental: RCT
Srichatrapimuk et al. *AIDS Res Ther.* 2023	n=24 PWH on ART, and with dyslipidemia Median age = 46 years	Pitavastatin vs placebo	Pitavastatin for 12 weeks vs placebo increased basic fibroblast growth factor, and decreased percentages of HLA-DR+CD38-CD4+ T cells and PD1+CD4+ T cells.	Experimental: Randomized Crossover Trial
Boczar et al. *J Nucl Cardiol Off Publ Am Soc Nucl Cardiol.* 2022	n=35 PWH, and HIV-ve control cohort	Rosuvastatin (n=17) vs usual care (n=18)	CD14^++^CD16^-^ and CCR2 expressions were reduced. Low-dose rosuvastatin significantly reduced bone marrow, spleen and thoracic aortic FDG uptake among PWH.	Experimental: RCT
Hearps et al. J Infect Dis, 2021	n=69 PWH on ART with moderate CVD riskMedian age = 54 years	Rosuvastatin (n=33) vs placebo (n=36)	No effect of rosuvastatin on IL-6, soluble tumor necrosis factor receptor type 2, CXCL10, sCD14 and soluble vascular cellular adhesion molecule 1 with increase in CD16+ monocytes.	Experimental: RCT
Trevillyan et al. AIDS, 2021	n=84 PWH on ART with moderate cardiovascular risk, Mean age = 54.1 years	Rosuvastatin (n=44) vsplacebo (n=40) for 96 weeks	No difference in progression of carotid artery intima-media thickness between two groups.	Experimental: RCT
Toribio et al. *AIDS* 2017	n=252 (Overall), PWH on ART, and with dyslipidemia Median age = 50 years	Pitavastatin and Pravastatin	sCD14, ox-LDL and Lp-PLA2 plasama levels reduced with pitavastatin vs pravastatin after 52 weeks	Experimental: RCT
Lipinski et al. Antivir Ther. 2020	n=147 ART-treated PWH Median age = 46 years 78% male	Rosuvastatin vs placebo	Rosuvastatin did not change volume, mass or regional distribution of coronary artery calcification	Experimental: RCT
Silverberg et al. *Ann Intern Med* 2009	n= 829 PWH and n = 6941 HIV- LDL and TG levels were elevated at baseline	Statin vs no statin	LDL levels reduced by 25.6% in PWH and by 28.3% in the other group (p=0.001)	Observational: Retrospective cohort
Funderburg et al. *JAIDS* 2015	n=147 ART treated PWH, Median age = 47 years	Rosuvastatin (n=72) vs placebo (n = 75)	Decrease in vascular inflammation and T-cell (CD4+CD38+HLA-DR+ and CD8+CD38+HLA-DR+) & monocyte activation (sCD14) after 48 weeks of rosuvastatin vs placebo	Experimental: RCT
Santos Junior et al. *Arq Bras Cardiol*. 2021	n=38 PWH on ART, and with low cardiovascular risk, Mean age = 42.6 years	Atorvastatin + Aspirin	Carotid intima-media thickness reduction was not associated with any of the evaluated risk factors	Observational: Case-Control
Lo et al. *Lancet HIV* 2015	n=40 PWH on ART with sub-clinical atherosclerosis	Atorvastatin (n=19) vs placebo (n=21)	Atorvastatin reduced non-calcified coronary plaque volume, number of high-risk coronary plaques, LDL and Lp-PLA2	Experimental: RCT

Dyslipidemia among PWH is characterized by elevated low density lipoproteins with a smaller particle size, decreased high density lipoproteins and hypertriglyceridemia (74). Of note, the smaller dense low-density lipoproteins are highly atherogenic, and are further intensified by the high level of triglycerides in circulation. Stopping Atherosclerosis and Treating Unhealthy bone with RosuvastatiN in HIV (SATURN-HIV) is the first double-blind RCT that studied markers of cardiovascular risk, skeletal health, and immune activation following statin therapy among ART-treated PWH (71). The study reported a reduction in several markers of inflammation and lymphocyte and monocyte activation after a 48-week rosuvastatin administration at a daily dose of 10mg (**Table 1**). Of note, the study found significant reductions in lipoprotein-associated phospholipase A2 (Lp-PLA2) which is a specific marker of vascular inflammation. Such observation further supports the non-lipid-lowering endothelial protective function of statins. Erlandson et al. conducted a secondary analysis of SATURN-HIV study and reported a worsening of insulin resistance in the statin group vs placebo from baseline to week 96 (75). However, the incidence of DM was similar in the two arms, which could be attributed to a relatively smaller sample size and a relatively short duration of the study. In contrast, HIV Outpatient Study (HOPS) reported an increased risk of incident DM with statin use among 4692 PWH which mirrored the findings in the general population (76). Such data suggest a risk-benefit consideration prior to statin use, especially in patients with a lower CVD risk.

While fluvastatin, rosuvastatin and pravastatin are generally considered safer statins, others such as simvastatin and atorvastatin interact with certain type of ART such as protease inhibitor (PI)-based regimens and may require dose adjustments or contraindication (77). PIs and other antiretrovirals such as efavirenz inhibit cytochrome P450 3A4 (CYP3A4) enzyme, which is predominantly required in the metabolism of statins. In addition, Malvestutto and colleagues showed pitavastatin devoid of interactions with contemporary ART including efavirenz (78). Among the newer ART classes, integrase strand transfer inhibitors (INSTIs) such as dolutegravir and bictegravir are preferred as a first-line therapy (79). INSTIs in general are not associated with unfavorable changes in lipids nor they are known to result in harmful interactions with statins (27, 79, 80). However, data from prospective RESPOND study of 17 European and Australin cohorts of about 30,000 PWH reported an increased risk of CVDs in the first 2 years of INSTI versus no-INSTI exposure after accounting for known CVD risk factors (80). Encouragingly, similar CVD risk levels were observed in the two groups beyond 2 years; thereby suggesting further confirmation of such results in long-term studies. In addition, the authors acknowledged the potential for unmeasured confounding and channeling bias influencing their study findings. Currently INSTI-based 2DR (such as dolutegravir+lamivudine) versus 3-DR are increasingly being employed which is not only a cost-effective strategy, but also reduces the burden of medications increasing compliance and reducing adverse effects while showing non-inferiority in several RCTs (21–23). Cahn et al. reported in secondary analysis of GEMINI-1 and GEMINI-2 RCTs with ART-naïve participants from 187 centres in 21 countries that 2DR dolutegravir+lamivudine was not inferior to 3DR through 144 weeks of the study period (22). Two cardiac related fatal adverse events in the 2DR group were not drug related, and the changes in lipids at 144 weeks from the baseline generally favored 3DR group. They further reported no differences in the levels of IL-6 and CRP at week 144 from baseline among the two groups. Similarly, Cossarizza et al. reported no differences in IL-6 levels between 2-DR and 3-DR arms in an open label RCT after 12 months of treatment switch (81). Contrastingly, Lombardi et al. showed decreased CRP levels after 1 year of treatment switch to 2-DR group versus treatment continuation in 3-DR group in a longitudinal analysis (23). Along these lines, data from SALSA RCT with ART-experienced virologically suppressed participants showed reduction in one (sCD14) out of four biomarkers of inflammation in the 2-DR versus 3-DR group over 48 weeks (82). The clinical significance of such change remains to be determined. On the other hand, TANGO RCT assessed random assignment to 2DR versus continuation of a tenofovir alafenamide (TAF)–based 3-or 4-drug regimen in ART-experienced virologically suppressed participants and showed no such benefits in inflammatory biomarkers (83, 84). However, their results favored INSTI-based 2DR in terms of overall changes in lipids such as total cholesterol, LDL and triglycerides. Taken together, more large-scale prospective assessments are required to determine the effect of INSTI based 2-DR in the reduction of inflammation and of CVD risk, which may further contribute in the reduction of clinical events. Such information would be useful as further guidance to the most correct place for statin administration among PWH.

Singh et al. reported on the strategy for the management of interactions which include prescription of appropriate statin and at a lower than recommended dose (85). However, the dose reduction has also been associated with reduced efficacy. On the other hand, a higher dose is linked with adverse effects such as a higher risk for the development of DM (86). Nearly 10% increase in the onset of DM following statin therapy has been reported in both observational and interventional studies (87, 88). A likely explanation of such increase in DM following statin therapy is owing to an increase in insulin resistance and/or β-cell dysfunction (88). Along these lines, risk-benefit analysis needs to be given a key consideration prior to statin administration among PWH owing to adverse effects such as DM. In 2019, the scientific committee of American Heart Association recommended preventive therapy among PWH with elevated CVD risk profile including presence of HIV-associated and/or more general factors such as a positive family history and chronic kidney disease (89). While REPRIEVE data strongly support statin use; others concluded a lower but non-significant decrease in the risk of all-cause mortality, any adverse effect and DM with statin treatment versus placebo/control (54, 90). Of note, study by Phan et al. included 127 participants and showed no difference in the progression of carotid artery intima-media thickness (cIMT) with statin use (54).

Riestenberg et al. have reported in an American cohort of 5,039 PWH and 10.011 controls that the former group was more likely to have ever taken pravastatin or atorvastatin (91). They further showed differences in statin prescription by ethnicity among PWH and such disparity was less pronounced among controls. Statins also provide benefits in decreasing chronic inflammation and immune activation which may decrease the risk for other non-AIDS events alongside CVDs (77).

## Statins as immune modulators: beyond their lipid-lowering effect

Beyond the lipid-lowering traditional role of statins, emerging studies have reported on their multifaceted effects ranging from anti-inflammatory immune modulatory functions (37), as briefly introduced earlier. They have been shown to inhibit NF-KB activity that further leads to a reduction of inflammation and of activated immune cells. Similarly, nitric oxide (NO) production is also increased by statins mainly via upregulation of endothelial NO synthase (eNOS) leading to a decrease in platelet aggregation and inflammation (33). Several mechanisms have been implicated in statin induced eNOS expression such as by activation of either the Phosphoinositide 3-kinase (PI3k)/Protein kinase B (Akt) pathway or the adenosine monophosphate (AMP)-activated protein kinase (AMPK) pathway or by the inhibition of the Rho/ROCK pathway.

Statins are implicated in both innate and adaptive immune response including in reduced T-cell activation (92, 93). They are reported to induce resistance of CD4 T cells to HIV-1 infection via upregulation of cyclin-dependent kinase inhibitor p21, together with inhibition of immune cell activation and proliferation *in vitro* (94). In the context of HIV infection, systemic immune activation further adds to the risk for CVDs thereby suggesting a pleiotropic role of statins (77). Two markers of immune activation have been extensively studied along these lines; 1) the soluble CD14 (sCD14), a circulatory marker of monocyte activation, also a surrogate marker of microbial translocation; and 2) the oxidized low-density lipoprotein (ox-LDL), a type of low density lipoprotein that may exacerbate atherosclerotic plaques and activate innate and adaptive immune responses (95). We and others have shown in PWH that sCD14 is linked with several markers of immune dysfunction (9, 14). Soluble CD14 is an indirect marker of microbial translocation which is related to lipopolysaccharide (LPS), a cell wall component of gram-negative bacteria that is a direct marker of microbial translocation (96). LPS is known to induce immune activation in chronic HIV infection (97) and has been inconsistently associated with clinical events (98–100). Such conflicting results could in part be explained by its low precision, association with serum lipid levels and methodological challenges encountered during its quantification, and potential cross-reactivity with βDG (17, 96, 101). LPS activates monocytes/macrophage via lipopolysaccharide binding protein together with toll-like receptor 4 resulting in the release of sCD14 (102, 103). Both sCD14 and ox-LDL have been associated with an increased risk for CVDs (103, 104). Encouragingly, statins have been shown to reduce the systemic level of both of these immune activation markers (20, 68, 105). Hileman et al. were the first to report a significant decrease in ox-LDL levels following a 10 mg daily dose of rosuvastatin versus placebo for 24-weeks among PWH on ART (106). Similarly, Nou et al. showed effectiveness of atorvastatin in decreasing ox-LDL levels among PWH on ART (107). A recent meta-analysis further reported variations in ox-LDL decrease with different types of statins (108). However, all statins analyzed were associated with a significant decrease in ox-LDL levels. Besides ox-LDL, Toribio et al. also attributed a reduction in sCD14 and Lp-PLA2 levels to pitavastatin vs pravastatin after 52 weeks of therapy (68). Of note, Lp-PLA2 is increasingly considered a novel biomarker for vascular wall inflammation.

Another emerging marker of cardiac dysfunction is soluble suppressor of tumorigenicity 2 (sST2), which is encoded by interleukin (IL)-1 receptor family gene ST2 (109, 110). Besides expressing the soluble isoform, ST2 gene also expresses a transmembrane ST2L isoform in a variety of cells such as dendritic cells, mast cells, macrophages, and T-helper cells (110, 111). The functional ligand of ST2 is the inflammation induced factor IL-33, an alarmin cytokine which is also released by myocardial endothelial cells. IL-33/ST2 axis plays an important role in acute and local inflammation as well as in issue repair. It is being increasingly employed as a prognostic marker in a variety of diseases including cardiac insufficiency and atherosclerosis. In a mouse model, Miller et al. (111) showed IL-33/ST2 signaling reduced the development of atherosclerosis. Secemsky et al. first reported sST2 to be linked with cardiac dysfunction and all-cause mortality among 332 PWH, of whom 79% were on long-term ART (15). We and others have shown sST2 to be associated with markers of inflammation and immune activation in the context of HIV infection (14). Interestingly, deFilippi et al. reported atorvastatin to be associated with a reduction in the levels of sST2 among PWH on ART which indicated a fibrosis mitigation effect (112). They observed a strong positive correlation of sST2 with sCD14 and monocyte chemoattractant protein 1 (MCP-1). Of note, the correlation between sST2 and sCD14 had previously been unearthed by our team (14). In addition, deFilippi et al. also reported a positive correlation between sST2 and ox-LDL (112), levels of both of these markers are reduced with statin use. Besides bacterial translocation from the gut to periphery, the translocation of fungal products such as the major cell wall component 1,3-β-D-Glucan (βDG), has also been shown to contribute to the chronic inflammation and immune activation and has been associated with cardiopulmonary dysfunction (17, 113, 114). We recently reported correlation of plasma βDG with markers of disease progression, gut damage, bacterial translocation, and inflammation among PWH (17). Whether statin use reduces βDG is yet to be explored, especially when statins are known to influence the systemic levels of markers of bacterial translocation to decrease the level of chronic inflammation and immune activation.

Besides translocation of microbial products, gut microbial dysbiosis has also been linked with chronic inflammation and immune activation and is not restored on long term ART (115–117), and thus could contribute to the risk for adverse events such as CVDs. Encouragingly, growing body of literature supports the notion that statins can regulate gut microbiome (118–120). On the other hand, gut microbiome has also been shown to impact statin efficacy by converting it into secondary metabolites as is the case with other medications. Such association may partly explain a compromised statin response and/or associated adverse effects in some patients (121, 122). Wilmanski et al. recently linked the composition of gut microbiome with heterogeneity of statin response (120). Notably, they showed more intense on/off target statin response in a diversity-depleted gut microbiome with abundance of Bacteroides. However, their study was limited by factors such as a cross-sectional study design and a pre-dominantly white study population from the US west coast and Western Europe.

Statins are implicated in the reduced prevalence of gut microbiota dysbiosis (118) and in the maintenance of homeostasis of gut microbiota by regulating intestinal innate immunity, exhibiting antibacterial activity and inhibiting cell membrane biosynthesis (119). In addition, statins influence bile acid metabolism that leads to changes in the composition of gut microbiota. Moreover, NF-κB signaling is also impacted by statins leading to changes in the composition of gut microbiota. Khan et al. reported in hypercholesterolemic patients an association of atorvastatin with relative abundance of anti-inflammation associated *Faecalibacterium prausnitzii*, *Akkermansia muciniphila*, and genus Oscillospira coupled with decreased abundance of proinflammatory Desulfovibrio species (123). Further studies evaluating the effects of statins on gut microbiota in the context of HIV-associated chronic inflammation and immune activation may highlight the therapeutic potential of these traditionally lipid-lowering agents. Therefore, targeting gut microbiota dysbiosis among PWH on ART represents a promising strategy for the reduction of CVDs (124).

In addition, statins are observed to be associated with cell growth, proliferation, secretion and apoptosis, bone formation and angiogenesis (125–127) (**Figure 2**). Such immune modulatory effects in conditions such as sepsis, pneumonia, influenza, cancer and HIV warrant further research to explore the pleiotropic role of statins (128–132).

## Conclusions and future directions

CVDs are known as the major contributor to morbidity and mortality among aging HIV-infected and uninfected populations. Such CVD risk is predicted to increase in the absence of targeted interventions to further jeopardize the quality of life of PWH, an infection which has evolved as a chronic disease. In the light of improvements in contemporary ART and guidelines suggesting prompt ART initiation following an HIV diagnosis, the quantification of CVD risk remains to be evaluated, followed by an assessment of need for interventions. Targeting traditional risk factors, particularly among PWH, with healthy life-style approaches such as smoking cessation and promotion of physical activity is of utmost importance, but may not be sufficient. Lipid-lowering statins can play a major role in the prevention of CVDs and such role remains less appreciated among PWH. Recent evidence suggests a broader implication of statins in the context of ART-treated HIV infection highlighted by a reduction in chronic inflammation and immune activation, which otherwise are fueled in part by microbial translocation and microbial dysbiosis owing to persistent gut damage. Thereby, ART-recipient PWH even with a low or median risk for CVDs can benefit from the pleiotropic effects of statins targeting not only dyslipidemia, but also chronic inflammation and immune activation. Along these lines, emerging areas include further elucidation of statin-gut microbiome interactions which may translate into a reduction of adverse events such as CVDs. The results from the large-scale, multi-centered, multi-national REPRIEVE study clearly show a more than expected reduction in the risk for major cardiovascular events with the use of pitavastatin. Such results can guide policy to lessen CVDs among PWH taking long-term ART.

## Author contributions

VM: Writing – review & editing, Writing – original draft, Conceptualization. JC: Writing – review & editing, Writing – original draft. JR: Writing – review & editing, Writing – original draft, Funding acquisition, Conceptualization.
